# Pulse pressure and all-cause mortality in ischaemic heart failure patients: a prospective cohort study

**DOI:** 10.1080/07853890.2022.2128208

**Published:** 2022-10-12

**Authors:** Weida Qiu, Xiaoju Xiao, Anping Cai, Zhiping Gao, Liwen Li

**Affiliations:** a The Second School of Clinical Medicine, Southern Medical University, Guangzhou, China; b Department of Cardiology, Guangdong Cardiovascular Institute, Guangdong Provincial People’s Hospital, Guangdong Academy of Medical Sciences, Guangzhou, China; c Concord Medical Center, Guangdong Provincial People’s Hospital, Guangdong Academy of Medical Sciences, Guangzhou, China

**Keywords:** Pulse pressure, blood pressure, ischaemic heart failure, left ventricular systolic dysfunction

## Abstract

**Background:**

Whether the association between pulse pressure (PP) and mortality varies with systolic blood pressure (SBP) in ischaemic heart failure (HF) with left ventricular systolic dysfunction (LVSD) is unknown.

**Objective:**

To evaluate the association between PP and all-cause mortality in ischaemic HF patients with SBP status at admission.

**Patients and methods:**

This prospective cohort study included 1581 ischaemic HF patients with LVSD. A total of 23.3% (*n* = 368) and 22.2% (*n* = 351) of the participants had SBP <110 mmHg and SBP >140 mmHg, respectively, with more than 80% of participants being male. Restricted cubic spline was performed to determine whether a nonlinear relationship existed between PP and all-cause mortality risk. A multivariable Cox proportional hazards model was used to assess the association between PP and all-cause mortality.

**Results:**

After a median of follow-up of 3.0 years, 257 events (16.4%) were observed in the cohort. There was a J-shaped relationship between PP and all-cause mortality (P value for nonlinearity = 0.020), with a risk nadir of approximately 46–49 mmHg. All-cause mortality risk varied with SBP status. Higher PP was associated with worse prognosis when the SBP was ≥110 mmHg, whereas the relationship did not reach statistical significance when the SBP was <110 mmHg.

**Conclusion:**

A J-shaped relationship between PP and all-cause mortality was observed in ischaemic HF patients with LVSD, and higher PP was associated with worse prognosis only in those with SBP ≥110 mmHg. Further studies are needed to corroborate these findings.

**KEY MESSAGES**
- A J-shaped relationship between pulse pressure and all-cause mortality was observed in ischaemic heart failure patients with left ventricular systolic dysfunction, with a risk nadir of approximately 46–49 mmHg.
- All-cause mortality risk varied with systolic blood pressure status, and higher pulse pressure was associated with worse prognosis when systolic blood pressure was above 110 mmHg.

## Introduction

Elevated pulse pressure (PP), a traditional indicator of aortic stiffening, has been regarded as a marker of poor outcome not only in healthy individuals [1,2] but also in those with hypertension and other comorbidities [3–6]. The prognostic value of PP in patients with heart failure (HF) is inconsistent. In HF with preserved ejection fraction (HFpEF) patients, both low and high PP were associated with adverse outcomes, suggesting a J-shaped relationship [7–9]. In HF with reduced EF (HFrEF) patients, PP showed a linear inverse relationship with mortality in clinical studies [9–11] and meta-analyses [12], whereas other studies displayed a J-shaped relationship [13]. Both of these findings were in contrast to findings of studies decades ago [14,15]. A potential explanation was that low PP, a marker of left ventricular systolic dysfunction (LVSD) and low stroke volume rather than aortic stiffening [10], was associated with worse outcomes in those with HFrEF. However, it has been noted that some HFrEF patients have systolic blood pressure (SBP) within the normal range despite reduced left ventricular ejection fraction (LVEF) [12], whereas others have a low SBP due to low stroke volume.

We herein hypothesize that the relationship between PP and mortality may vary with SBP status in HFrEF patients. In this study, we assessed the association between PP and all-cause mortality according to SBP status in ischaemic HF patients with LVSD.

## Methods

### Study design and participants

This was a single-centre, prospective cohort study including ischaemic HF patients with LVSD as described previously [16]. In brief, ischaemic aetiology of HF was defined based on coronary angiography, a prior history of myocardial infarction (MI), or prior revascularization including percutaneous coronary intervention (PCI) or coronary artery bypass grafting (CABG). LVEF was assessed during the index hospitalization using echocardiogram and those with LVEF <45% were eligible for this study. Participants aged >18 years who were hospitalized in the Department of Cardiology, Guangdong Provincial People’s Hospital from December 2015 to June 2019 were screened. Patients with HF due to non-ischaemic aetiology (e.g. idiopathic dilated cardiomyopathy, valvular heart disease) or patients who were lost to follow-up since discharge were excluded. This study was approved by the Clinical Research Ethics Committee of Guangdong Provincial People’s Hospital (approval reference number: GDREC2017172H) and was performed in accordance with the Declaration of Helsinki. Before enrolment, all patients provided written informed consent.

### Study procedure

Baseline data of interest were extracted from the electronic medical records of Guangdong Provincial People’s Hospital, including demographics, the reason for admission, vital signs at admission, comorbid conditions, echocardiographic parameters and medical therapy at discharge. As reported previously [16], fasting venous blood was drawn to evaluate lipid parameters and haemoglobin A1c (HbA1c) levels on the second day after admission. Venous blood at admission was drawn to evaluate creatinine, high-sensitivity C-reactive protein (hs-CRP), high-sensitivity cardiac troponin-T (hs-cTnT) and N-terminal pro-brain natriuretic peptide (NT-proBNP) levels. The estimated glomerular filtration rate (eGFR) was calculated using the modified diet of renal disease formula using serum creatinine [17], and an eGFR <60 ml/min/1.73 m^2^ was defined as chronic kidney disease (CKD).

### Blood pressure measurement

After the participants had rested and sat quietly for 5 min, brachial BP was measured at admission by experienced physicians or nurses using electronic sphygmomanometers, and PP was defined as the difference between the SBP and diastolic BP (DBP). Patients were classified into three groups based on the SBP at admission: the low SBP group (<110 mmHg), normal SBP group (110–140 mmHg) and high SBP group (>140 mmHg).

### Echocardiographic examination

Transthoracic echocardiography was performed by experienced sonographers when the patient’s clinical condition was stable. The LVEF was measured using the modified biplane Simpson’s rule from the apical 2- and 4- chamber view. Left atrial and left ventricular sizes were measured by 2D echocardiography. Mitral inflow E- and A-wave velocity was assessed using pulsed wave Doppler from the apical 4-chamber view. Peak early diastolic tissue velocity (e′) was measured from the septal aspect of the mitral annulus and estimated left ventricular (LV) filling pressure was calculated using the E/e′ ratio [18].

### Follow-up and clinical outcomes

Follow-up was performed through phone call interviews. Due to the unavailability of assessment for the exact reason of death, all-cause mortality was used in the current study. The follow-up time was calculated as the date of death or last follow-up minus the date of discharge.

### Statistical analysis

Continuous variables were expressed as the mean value ± standard deviation (*SD*) or median (interquartile range), and categorical variables were presented as numbers and proportions. Student’s *t*-test or the Kruskal–Wallis H-test was used to compare continuous variables, and the Chi-square test was used to compare categorical variables. Differences in baseline characteristics were examined among the three SBP groups.

To assess whether there was a nonlinear relationship between PP and all-cause mortality, restricted cubic spline was performed. The rate of all-cause mortality according to quintiles of PP was displayed in a dot plot.

The cumulative incidence of all-cause mortality was plotted with the Kaplan–Meier (KM) curve and was compared between low and high PP subgroups stratified by 3 SBP groups with the log-rank test. The hazard ratio (HR) and 95% confidence interval (CI) were computed using the Cox proportional-hazards analyses, with stepwise adjustment for covariates, including age, sex, the reason for admission, New York Heart Association (NYHA) class, smoking status, diabetes, dyslipidemia, eGFR, low-density lipoprotein cholesterol (LDL-C), log10 hs-cTNT, log10 NT-proBNP, left ventricular end diastolic diameter (LVEDD), left ventricular end systolic diameter (LVESD), LVEF, left atrial (LA) diameter, the E/e′ ratio, the use of intravenous diuretics and intravenous inotropic agents. Missing values for hs-cTnT, NT-proBNP, eGFR, LDL-C and LA diameter were imputed with the median, whereas covariates with >10% missing data (e.g. hs-CRP) were excluded.

Two-sided *p* values < .05 were considered statistically significant. All analyses were performed using Stata version 15.1 (StataCorp LLC, College Station, TX, USA) and R version 4.1.1 (The R Project for Statistical Computing, Vienna, Austria).

## Results

### Baseline characteristics

**Figure 1.**
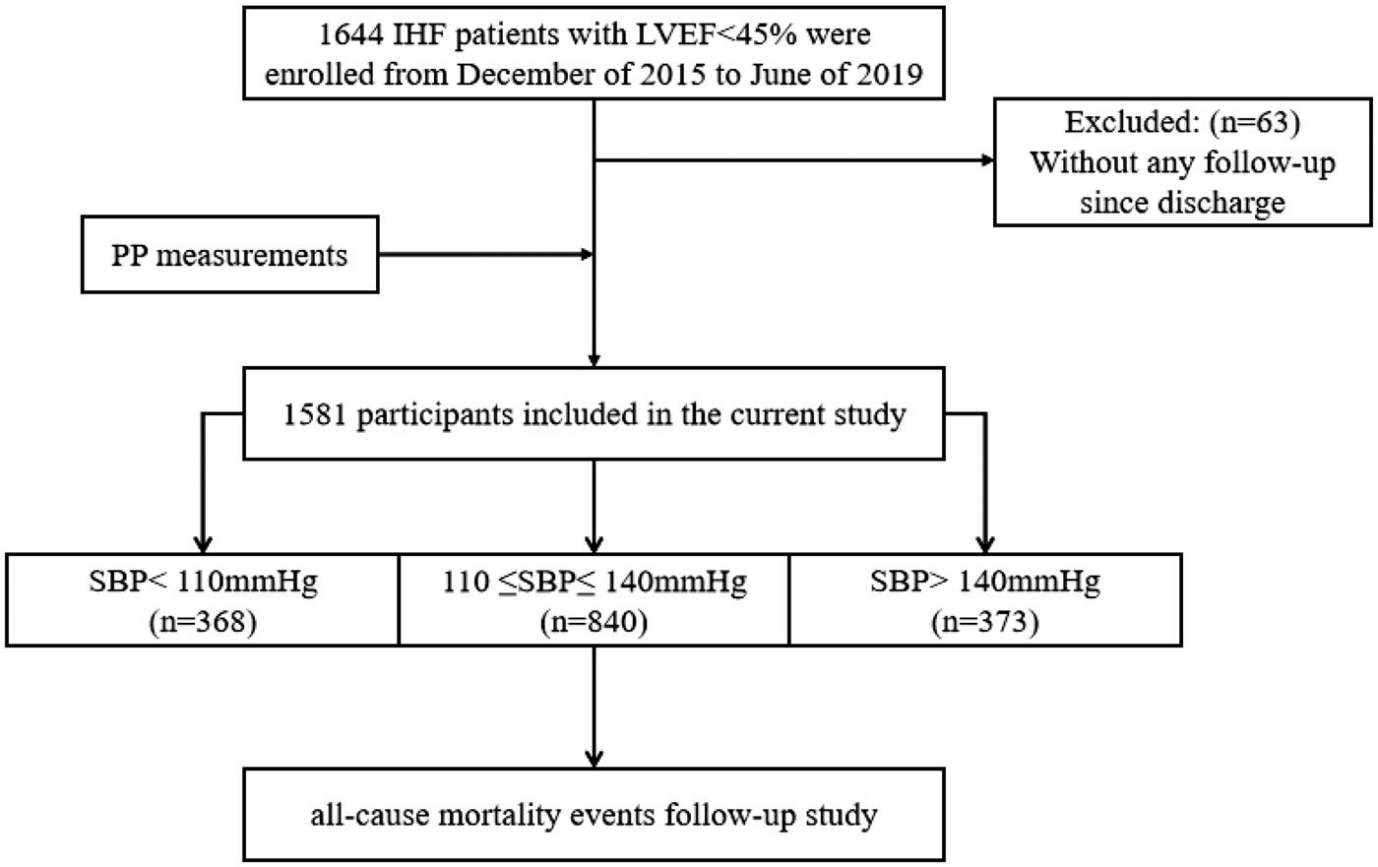
Study flowchart. IHF: ischaemic heart failure; LVEF: left ventricular ejection fraction; PP: plus pressure; SBP: systolic blood pressure.

A total of 1644 patients with ischaemic HF between December 2015 and June 2019 were recruited, and 1581 were enrolled in this study (Figure 1). Table 1 shows the baseline differences among the three SBP groups. Briefly, patients with SBP >140 mmHg were older, had higher DBP, PP and LVEF values and were less likely to present with ST segment elevation myocardial infarction (STEMI). Patients with SBP <110 mmHg had higher hs-cTnT and NT-proBNP levels and were more likely to receive intravenous inotropic agents and diuretics. The comorbidities (except for anaemia and stroke/transient ischaemic attack, TIA) were comparable between the three SBP groups.

**Table 1.** Baseline characteristics comparison between systolic blood pressure group.

Variables	SB*p* < 110 mmHg (*n* = 368)	110 ≤ SB*p* ≤ 140 mmHg (*n* = 862)	>140 mmHg (*n* = 351)	*p*-Value
Age (years)	61.1 ± 11.4	63.3 ± 10.9	65.7 ± 10.3	<.001
Male, *n* (%)	310 (84.2)	750 (87.0)	287 (81.8)	.055
Vital sign at admission				
Systolic blood pressure (mm Hg)*	102 (96–106)	124 (118–131)	150 (145–158)	<.001
Diastolic blood pressure (mm Hg)*	64 (58–69)	74 (69–81)	85 (77–92)	<.001
PP (mmHg)*	37 (32–42)	49 (42–57)	67 (58–77)	<.001
Heart rate (beat per minute)*	79 (70–91)	78 (70–88)	78 (70–90)	.198
Reason for admission				
STEMI, *n* (%)	83 (22.6)	119 (13.8)	30 (8.6)	<.001
Non-STEMI, *n* (%)	32 (8.7)	58 (6.7)	30 (8.6)	.366
Unstable angina, *n* (%)	178 (48.4)	454 (52.7)	196 (55.8)	.130
Acute heart failure, *n* (%)	136 (37.0)	329 (38.2)	141 (40.2)	.668
NYHA Class III–IV, *n* (%)	99 (26.9)	218 (25.3)	97 (27.6)	.658
Comorbidities				
Current smoker, *n* (%)	84 (22.8)	211 (24.5)	78 (22.2)	.650
Diabetes mellitus, *n* (%)	128 (34.8)	278 (32.3)	125 (35.6)	.456
Dyslipidemia, *n* (%)	229 (62.2)	564 (65.4)	236 (67.2)	.353
Chronic heart failure, *n* (%)	152 (41.3)	348 (40.4)	144 (41.0)	.947
Anaemia, *n* (%)	98 (26.6)	146 (16.9)	78 (22.2)	<.001
CKD, *n* (%)	84 (22.8)	198 (23.0)	94 (26.8)	.326
COPD, *n* (%)	28 (7.6)	58 (6.7)	31 (8.8)	.440
Atrial fibrillation, *n* (%)	17 (4.6)	42 (4.9)	12 (3.4)	.536
Prior stroke/TIA, *n* (%)	15 (4.1)	68 (7.9)	43 (12.3)	<.001
Prior MI, *n* (%)	140 (38.0)	321 (37.2)	105 (29.9)	.032
Prior PCI, *n* (%)	225 (61.1)	500 (58.0)	187 (53.3)	.099
Prior CABG, *n* (%)	4 (1.1)	19 (2.2)	7 (2.0)	.416
Laboratory				
Haemoglobin (g/L)*	130 (117–143)	134 (122–146)	134 (119–145)	.012
Total cholesterol (mmol/L)*	4.10 (3.32–5.00)	4.20 (3.52–5.00)	4.30 (3.60–5.10)	.022
LDL-C (mmol/L)*	2.67 (2.14–3.33)	2.73 (2.20–3.33)	2.79 (2.25–3.41)	.140
HDL-C (mmol/L)*	0.87 (0.73–1.03)	0.92 (0.79–1.06)	0.96 (0.81–1.11)	<.001
Triglyceride (mmol/L)*	1.27 (0.95–1.76)	1.28 (1.00–1.81)	1.31 (0.98–1.77)	.559
Lp (a) (mg/dL)*	21.8 (10.4–46.2)	18.5 (9.8–39.0)	22.2 (10.2–43.6)	.194
Creatinine (µmol/L)*	93.9 (80.5–108.0)	90.9 (77.7–108.9)	91.0 (75.1–111.2)	.572
eGFR (ml/min/1.73 m^2^)*	74.3 (61.2–88.3)	75.5 (61.3–91.3)	74.1 (57.9–89.3)	.264
HbA1c (%)*	6.2 (5.7–7.4)	6.3 (5.8–7.5)	6.4 (5.8–7.6)	.222
FPG (mmol/L)*	5.31 (4.61–6.89)	5.35 (4.64–6.74)	5.49 (4.62–6.82)	.814
Hs-CRP (mg/L)*	5.56 (1.40–19.26)	5.30 (1.33–16.30)	4.61 (1.63–11.4)	.492
Hs-cTNT (pg/mL)*	58.3 (22.7–430.8)	34.6 (17.6–188.3)	29.3 (16.5–100.8)	<.001
NT-proBNP (pg/mL)*	2163 (991–4447)	1410 (551–3488)	1473 (549–3291)	<.001
Echocardiographic index				
LA diameter (mm)*	40 (36–45)	40 (36–44)	40 (37–44)	.557
LVESD (mm)*	48 (41–54)	47 (41–53)	46 (40–52)	.054
LVEDD (mm)*	59 (53–65)	59 (53–64)	58 (53–63)	.332
LVEF (%)*	35 (29–39)	36 (30–41)	38 (32–41)	<.001
E/e*	16.7 (12.5–25.0)	16.2 (12.1–22.3)	16.3 (12.8–22.0)	.182
Coronary angiography				
LM, *n* (%)	88 (23.9)	218 (25.3)	90 (25.6)	.841
LAD, *n* (%)	331 (90.0)	793 (92.0)	323 (92.0)	.463
LCX, *n* (%)	253 (68.8)	635 (73.7)	268 (76.4)	.062
RCA, *n* (%)	277 (75.3)	664 (77.0)	278 (79.2)	.454
Single vessel, *n* (%)	49 (13.3)	105 (12.2)	45 (12.8)	.850
Two vessels, *n* (%)	76 (20.7)	173 (20.1)	64 (18.2)	.687
Three vessels, *n* (%)	220 (59.8)	547 (63.5)	232 (66.1)	.208
In-hospital treatment				
IV inotropic agents, *n* (%)	80 (21.7)	100 (11.6)	26 (7.4)	<.001
IV diuretics, *n* (%)	160 (43.5)	292 (33.9)	123 (35.0)	.005
Coronary stenting, *n* (%)	245 (66.6)	586 (68.0)	235 (67.0)	.870
Medications at discharge				
Aspirin, *n* (%)	317 (86.1)	767 (89.0)	311 (88.6)	.357
Clopidogrel, *n* (%)	264 (71.7)	632 (73.3)	267 (76.1)	.409
Ticagrelor, *n* (%)	58 (15.8)	114 (13.2)	33 (9.4)	.038
Statins, *n* (%)	345 (93.8)	827 (95.9)	330 (94.0)	.171
Betablocker, *n* (%)	311 (84.5)	727 (84.3)	297 (84.6)	.992
RASi, *n* (%)	207 (56.3)	625 (72.5)	280 (79.8)	<.001
ARNI, *n* (%)	10 (2.7)	24 (2.8)	12 (3.4)	.811
MRA, *n* (%)	213 (57.9)	439 (50.9)	158 (45.0)	.003
Loop diuretic, *n* (%)	200 (54.4)	405 (47.0)	160 (45.6)	.030
Digoxin, *n* (%)	33 (9.0)	71 (8.2)	14 (4.0)	.018
CCB, *n* (%)	4 (1.1)	68 (7.9)	86 (24.5)	<.001
Insulin, *n* (%)	31 (8.4)	55 (6.4)	25 (7.1)	.437
Oral anti-diabetics, *n* (%)	121 (32.9)	259 (30.1)	106 (30.2)	.596
Oral anticoagulants, *n* (%)	35 (9.5)	63 (7.3)	24 (6.8)	.325

PP: pulse pressure; STEMI: ST segment elevation myocardial infarction; non-STEMI: non-ST segment elevation myocardial infarction; CKD: chronic kidney disease; COPD: chronic obstructive pulmonary disease; TIA: transient ischaemic attack; MI: myocardial infarction; CABG: coronary artery bypass grafting; LDL-C: low density lipoprotein-cholesterol; HDL-C: high density lipoprotein cholesterol; Lp(a): lipoprotein(a); eGFR: estimated glomerular filtration rate; HbA1c: glycated haemoglobin A1c; FPG: fasting plasma glucose; Hs-CRP: high sensitivity C reactive protein; Hs-cTnT: high sensitivity cardiac troponin-T; NT-proBNP: N-terminal pro B-type natriuretic peptide; LA: left atrial; LVESD: left ventricular end systolic diameter; LVEDD: left ventricular end diastolic diameter; LVEF: left ventricular ejection fraction; IV: intravenous; LM: left main coronary artery; LAD: left anterior descending coronary artery; LCX: left circumflex artery; RCA: right coronary artery; RASi: renin-angiotensin-system inhibitor; ARNI: angiotensin receptor-neprilysin inhibitor; MRA: mineralocorticoid receptor antagonist; CCB: Calcium channel blocker; *presented as median (interquartile range).

### Relationship of pulse pressure with all-cause mortality

**Figure 2.**
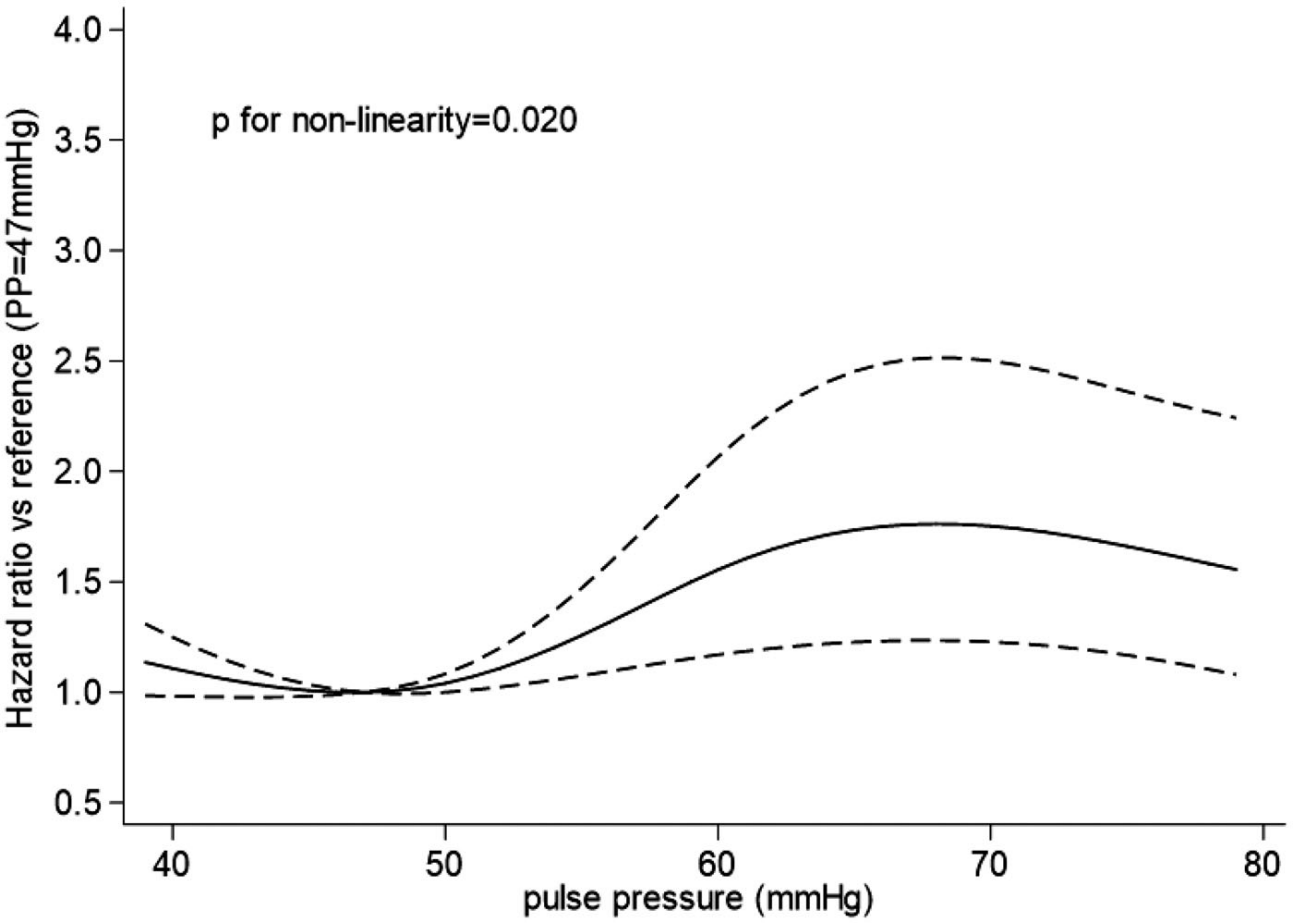
Association between pulse pressure and risk of all-cause mortality.

**Figure 3.**
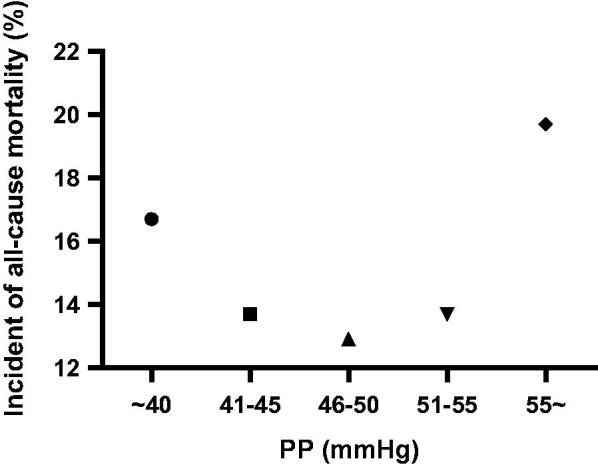
Incident rate of all-cause death events according to groups defined by quintiles of pulse pressure.

The crude all-cause mortality rate was 16.4% (*n* = 259). Restricted cubic spline showed a nonlinear (J-shaped) relationship between PP and all-cause mortality (*p* value for nonlinearity = 0.020), with a risk nadir of approximately 46–49 mmHg (Figure 2). The rate of all-cause mortality according to the quintiles of PP is shown in Figure 3. Both PP >50 mmHg and <46 mmHg were associated with an increased risk of all-cause mortality.

### All-cause mortality by median PP value according to SBP

In the SBP <110 mmHg group, the cumulative incidence of all-cause mortality was similar in patients with a PP below and above the median PP (20.3% vs. 15.7%; *p* = .230), which was consistent when PP was considered a continuous variable. In the SBP between 110 and 140 mmHg group and in the SBP >140 mmHg group, patients with PP above the median had a higher all-mortality risk, with adjusted HRs of 1.54 (95%CI 1.08–2.20) and 2.00 (95% CI 1.15–3.48), respectively. When PP was considered a continuous variable, in the SBP of 110 to 140 mmHg group, all-cause mortality risk increased with increased PP ((HR 1.20 and 95% CI 1.03–1.40) per 10-mmHg increase in PP), whereas in the SBP >140 mmHg group, there was a trend towards increased all-cause mortality risk with increased PP (*p* = .097) (Table 2, Figure 4).

Figure 4.Kaplan–Meier curve of all-cause mortality stratified by systolic blood pressure according to pulse pressure level. (a) SBP >140 mmHg group. (b) 110 ≤ SBP ≤140 mmHg group. (c) SBP <110 mmHg group.

**Figure:**
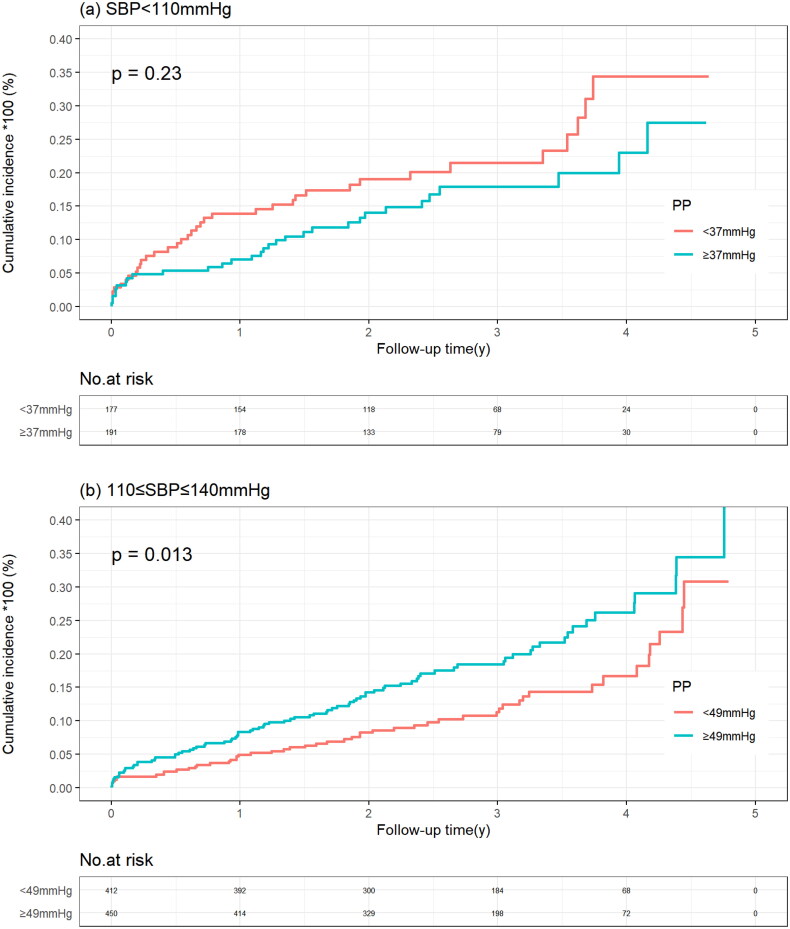

**Figure:**
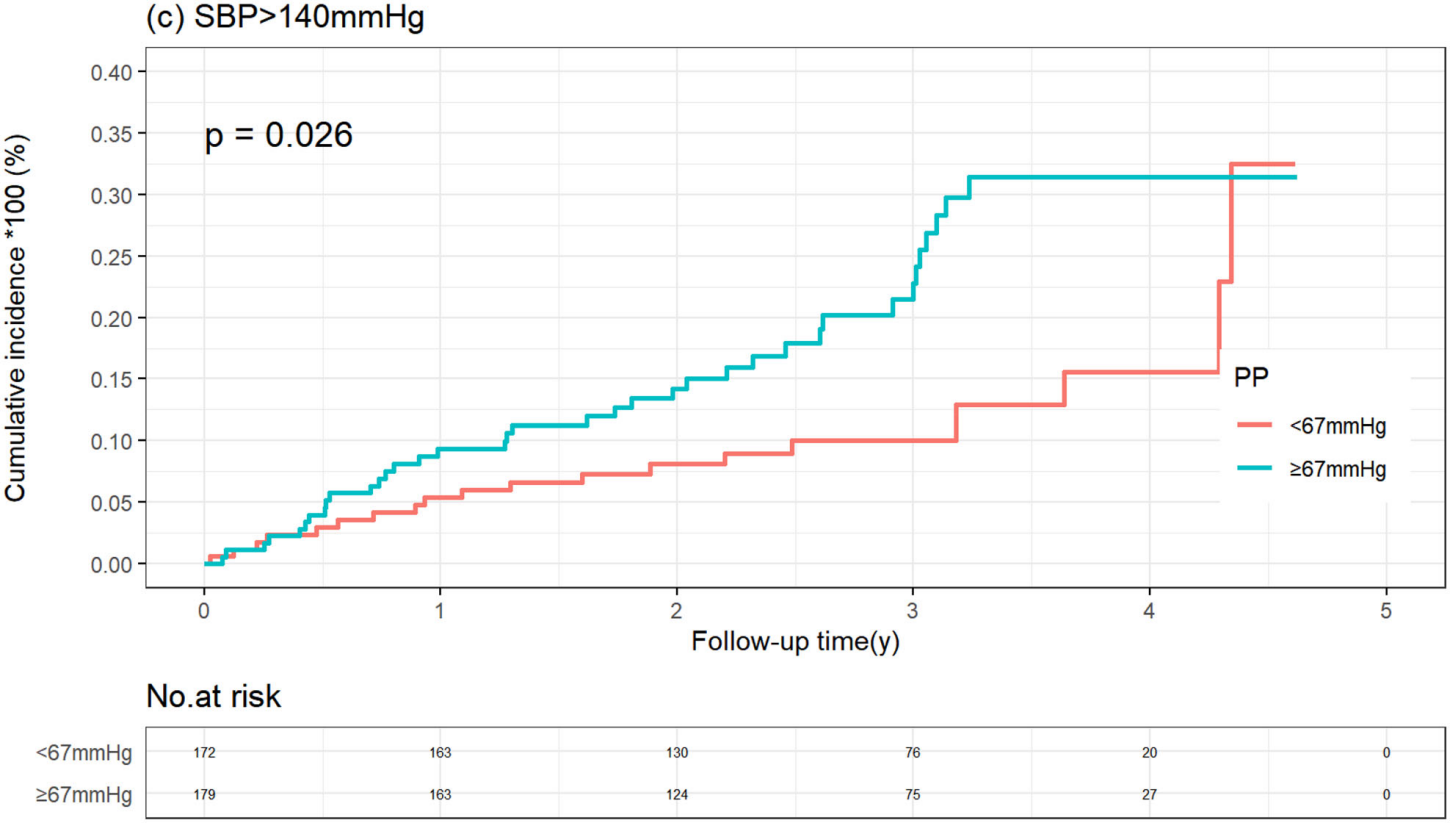

**Table 2.** PP level-associated all-cause mortality risk according to SBP groups.

Groups	Events/total (%)	HR (95% CI)	*p* Value	Adjusted HR (95% CI)	*p*-Value
**SBP <110 mmHg**	**66/368 (17.9)**				
Per 10-mmHg PP increase	66/368 (17.9)	0.85 (0.62–1.15)	.283	/	/
PP <37 mmHg	36/177 (20.3)	Reference	.230	Reference	/
PP ≥37 mmHg	30/191 (15.7)	0.74 (0.46–1.20)		/	
**110 ≤ SBP ≤140 mmHg**	**136/862 (15.8)**				
Per 10-mmHg PP increase	136/862 (15.8)	1.18 (1.02–1.38)	.027	1.20 (1.03–1.40)	.019
PP <49 mmHg	51/412 (12.4)	Reference	.013	Reference	.018
PP ≥49 mmHg	85/450 (18.9)	1.56 (1.10–2.21)		1.54 (1.08–2.20)	
**SBP >140 mmHg**	**57/351 (16.2)**				
Per 10-mmHg PP increase	57/351 (16.2)	1.22 (0.99–1.56)	.068	1.34 (0.97–1.76)	.097
PP <67 mmHg	20/172 (11.6)	Reference	.026	Reference	.014
PP ≥67 mmHg	37/179 (20.7)	1.84 (1.07–3.32)		2.00 (1.15–3.48)	

Multivariable Cox proportional hazards regression model was adjusted for age, sex, reason for admission, NYHA class, smoking status, diabetes, dyslipidemia, eGFR, LDL-C, log10 Hs-cTNT, log10 NT-proBNP, LVEDD, LVESD, LVEF, LA diameter, E/e′, intravenous diuretics, intravenous inotropic agents.

## Discussion

The results of this study demonstrated a J-shaped relationship between PP and all-cause mortality in ischaemic HF patients with LVSD, and this relationship varied by SBP status. Specifically, when the SBP was ≥110 mmHg, an increased PP was associated with a higher all-cause mortality risk, whereas when the SBP was <110 mmHg, the association between increased PP and all-cause mortality was nonsignificant.

Consistent with a previous report [13], our current study also showed a J-shaped relationship between PP and all-cause mortality in ischaemic HF patients with LVSD. Interestingly, the relationship between PP and all-cause mortality risk varied by baseline SBP status in the present study. PP has been considered a complex marker in the HF population [19], and the explanations for the present study are speculative. Previous studies have shown that a low PP was associated with increased mortality in advanced HF patients, while the trend was opposite in asymptomatic HFrEF patients [20]. In the current study, patients in the SBP <110 mmHg group had higher NT-proBNP values and were more likely to receive intravenous diuretics and inotropic agents than their counterparts in the SBP ≥110 mmHg group, suggesting that patients in the low SBP group (SBP <110 mmHg) might be more likely to have advanced HF. This might explain why high PP was related to death in the normal (110–140 mmHg) and high (>140 mmHg) SBP groups. However, no association was observed between PP and all-cause mortality in the low SBP group, which was inconsistent with previous study [20]. This discrepant finding might be due to the small sample size or different clinical characteristics of the participants in our current study.

Notably, SBP is associated with left ventricular systolic function [21]. We speculated that a high PP was mainly due to arteriosclerosis in the normal SBP group, leading to a high mortality risk [22,23], whereas a low PP in the low SBP group, which reflected the severity of LV systolic dysfunction, played a critical role in the prognosis [10]. Importantly, the median PP in the low SBP group in the current study was far below than that in other study of the relationship between PP and atherosclerosis [24]. Previous studies have also confirmed that aortic stiffness is more common in individuals with hypertension than in the normotensive population [25,26], suggesting that hypertensive patients with a high PP are at a higher mortality risk due to arterial stiffness [22].

In accordance with the MAGGIC (Meta-Analysis Global Group in Chronic Heart Failure) analysis in HFrEF patients [12], the current study also showed that when patients had SBP >140 mmHg, higher PP was associated with a worse prognosis. The results of our study extend prior studies to assess the relationship between PP and all-cause mortality in ischaemic HF patients with normal SBP. The findings suggest that the all-cause mortality risk increases at a higher PP level even in those with controlled SBP. Combined with the recent study [24], it is considered that PP should be taken into consideration not only in patients with concomitant hypertension, but also in those who are at target SBP.

In contrast to the present study, Bonapace S and co-workers found that the risk of all-cause mortality at 1 year decreased with high PP in acute HFrEF patients with SBP between 100 and 140 mmHg [9]. The explanation is as follows. First, participants involved in their study were de novo and worsening HF patients, including 88% of patients who were in NYHA class III or IV [9], which was higher than our current study. As discussed above, advanced HF patients with low PP usually have poor prognosis [20]. Second, the prevalence of atrial fibrillation in Bonapace S’ study was much higher than that in ours. This may be another reason to explain the discrepancy because loss of atrial contribution to LV filling caused an obvious reduction in stroke volume [27].

### Study limitations

The current study has several limitations. First, this was a single-centre cohort study so the conclusions of the study may not be generalizable to all ischaemic HF populations. Second, observation studies have inherent limitations, including missing values and incomplete information, the collection of nonrandomized data, and potential unknown confounding factors. Third, we only evaluated PP at admission, and the alterations in PP at discharge or during follow-up were unknown. Nevertheless, PP amplitude varies by time [28], and data for PP collected early at admission were related to poor prognosis [29]. Whether variation in PP affects prognosis in ischaemic HF populations should be examined in future studies. Fourth, all-cause mortality was used as the endpoint because we were unable to ascertain the cause of death through phone call interviews. The association between PP and cardiovascular mortality or HF hospitalization in the ischaemic HF population should be further studied.

## Conclusion

There was a J-shaped association between PP and all-cause mortality in ischaemic HF patients with LVSD, and the risk of mortality varied with SBP status. A higher PP was associated with worse prognosis in those with SBP > 110 mmHg, whereas the relationship did not reach statistical significance in those with low SBP. Further large randomized clinical trials are warranted to confirm our current results.

## Data Availability

The deidentified participant data will be shared on a request basis. Please directly contact the corresponding author to request data sharing.

## Permission to reproduce material from other sources

All the authors permit this manuscript has not been published or presented elsewhere in part or in entirety and not under consideration by another journal.
